# ANGPTL4/8 promotes plasmin-mediated cleavage of LPL inhibitors

**DOI:** 10.1016/j.jlr.2023.100438

**Published:** 2023-09-09

**Authors:** Sander Kersten

**Affiliations:** 1 Nutrition, Metabolism and Genomics Group, Division of Human Nutrition and Health, Wageningen University, Wageningen, The Netherlands; 2 Division of Nutritional Sciences, Cornell University, Ithaca, NY, USA

The key function of adipose tissue is to store excess energy as triglycerides. After digestion and absorption in the gastrointestinal tract, dietary triglycerides are shuttled to the adipose tissue packaged in chylomicron particles. The triglycerides in chylomicrons are hydrolyzed by the enzyme LPL, which is bound to the surface of capillaries in the adipose tissue. Seminal studies performed over 6 decades ago showed that the activity of LPL in adipose tissue depends on the nutritional status, with the highest LPL activity found in the fed state and the lowest after prolonged fasting (1). The high LPL activity after a meal promotes the efficient clearance of dietary triglycerides from the circulation for storage in adipose tissue.

A key observation made by the team of Olivecrona about 20 years ago was that the downregulation of LPL activity during fasting requires that a gene, separate from *LPL*, is turned on (2). Converging evidence led to the realization that this gene encodes the secreted factor ANGPTL4, which was cloned 2 years earlier and was initially named fasting-induced adipose factor (3, 4, 5). ANGPTL4 is a potent inhibitor of LPL in vitro and in vivo (6). Biochemical studies have shown that ANGPTL4 causes the irreversible unfolding of LPL, although other mechanisms have been proposed (7). In vivo, the interaction between ANGPTL4 and LPL leads to the degradation of LPL, thereby reducing the capacity for clearing circulating triglycerides in the capillaries of adipose tissue (7).

ANGPTL4 is part of a subfamily of angiopoietin-like proteins that also include ANGPTL3 and ANGPTL8. ANGPTL3 was discovered as a liver-derived LPL inhibitor that is mainly active in the fed state (8, 9). For many years, two questions remained unanswered. First, why is ANGPTL3 a much weaker LPL inhibitor than ANGPTL4 when studied in vitro? This observation did not match with the marked impact of ANGPTL3 inactivation or overexpression on plasma triglycerides in vivo (10). Second, how does ANGPTL3 exclusively regulate LPL in the fed state, even though its production in the liver is not regulated by nutritional status (11)?

This is where ANGPTL8 welcomingly entered the scene about a decade ago. ANGPTL8 is a shortened member of the ANGPTL family whose production in the liver and adipose tissue is extremely sensitive to feeding (upregulation) and fasting (downregulation). It is now well established that ANGPTL3 forms a complex with ANGPTL8 in the liver and that this ANGPTL3/8 complex is the dominant physiological inhibitor of LPL in the fed state (12, 13, 14). Two outstanding questions remained, though. First, why is ANGPTL8 also produced in the adipose tissue in addition to the liver? Adipose tissue does not produce any ANGPTL3. Second, how it is possible that in the fed state, the ANGPTL3/8 complex promotes the preferential uptake of circulating triglycerides in white adipose tissue rather than skeletal muscle, heart, and brown adipose tissue (15)?

Several articles published in the past few years, culminating with the article by Chen in this issue of *JLR*, have revealed an elaborate mechanism revolving around a complex between ANGPTL4 and ANGPTL8 (16, 17, 18). This ANGPTL4/8 complex is formed in adipocytes and, unlike the ANGPTL3/8 complex, acts locally to promote the clearance of circulating triglycerides, as indicated by the elevated plasma triglycerides and reduced postheparin plasma LPL content in mice with adipocyte-specific deficiency of ANGPTL8 (18). How does ANGPTL8 do that? First, the ANGPTL4/8 complex, by strongly binding but only weakly inhibiting LPL, shields LPL from strong inhibition by ANGPTL3/8 (Fig. 1) (16, 17). Second, ANGPTL8 may reduce the secretion of ANGPTL4 by adipocytes, possibly by promoting the intracellular degradation of ANGPTL4 (18). Two recent articles by the group of Robert Konrad at Eli Lilly have added a surprising new twist. They found that the ANGPTL4/8 complex binds tissue plasminogen activator (tPA) and plasminogen, the precursor of the fibrinolytic enzyme plasmin (19). Activation of tPA by ANGPTL4/8 subsequently leads to the generation of plasmin. In the first article, it was proposed that the ANGPTL4/8-induced plasmin formation causes cleavage and inactivation of ANGPTL4/8 itself, suggesting a negative feedback loop (19). However, the physiological rationale for such a pathway remained elusive. In their latest article, Konrad *et al.* now show that the ANGPTL4/8-induced plasmin formation causes the degradation of the LPL inhibitors ANGPTL4, ANGPTL3, and ANGPTL3/8 (20).

**Fig. 1:**
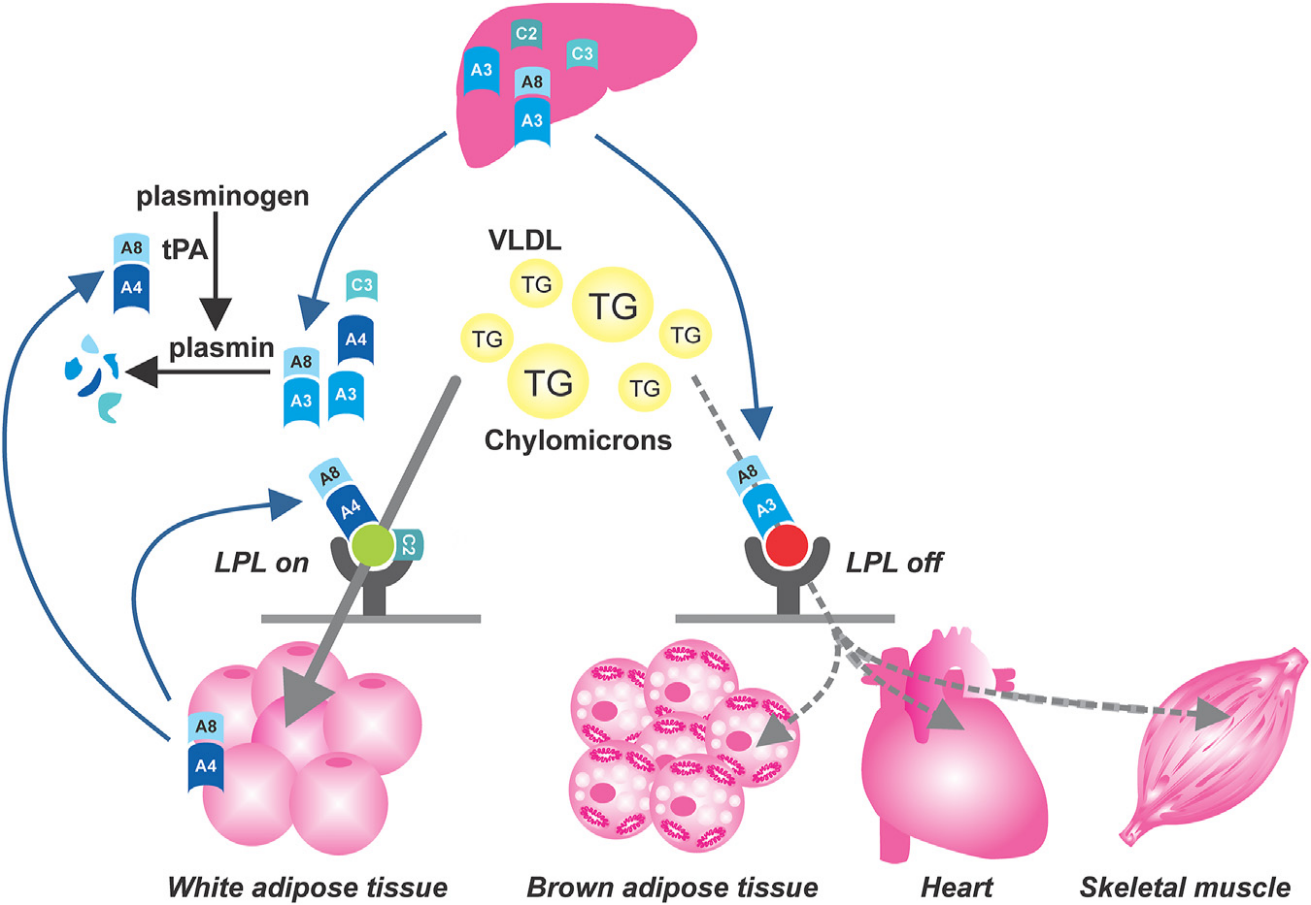
Model for the mode of action of ANGPTL4/8 in adipose tissue in the fed state. Several modes of action have been proposed for ANGPTL4/8 in white adipose tissue, all contributing to attenuating the local inhibition of LPL by ANGPTL3/8 and ANGPTL4. First, the ANGPTL4/8 complex, by strongly binding but only weakly inhibiting LPL, shields LPL from strong inhibition by ANGPTL3/8. Second, ANGPTL8 may reduce the secretion of ANGPTL4 by adipocytes, possibly by promoting the intracellular degradation of ANGPTL4 (not shown). Third, ANGPTL4/8 activates tPA to promote the formation of plasmin. Plasmin in turn causes the degradation of the LPL inhibitors ANGPTL4, ANGPTL3, ANGPTL3/8, and APOC3. The LPL activator APOC2 is refractory to degradation by plasmin. As the ANGPTL4/8 complex is only produced in adipose tissue and acts locally in an autocrine manner, the above mechanisms are exclusive to adipose tissue. In other tissues, the liver-derived ANGPTL3/8 complex inhibits LPL, thereby ensuring the selective clearance of circulating triglycerides in adipocyte tissue for storage. Please note that APOC2 and APOC3 are normally bound to triglyceride-rich lipoproteins. A3, ANGPTL3; A4, ANGPTL4; A8, ANGPTL8; C2, apolipoprotein C2; C3, apolipoprotein C3; TG, triglyceride.

For their studies, Chen *et al.* used LPL stable expressing cells to which they added either ANGPTL3/8, ANGPTL4, or ANGPTL3, combined with tPA/plasminogen and ANGPTL4/8. Besides promoting the degradation of ANGPTL3/8, ANGPTL4, and ANGPTL3, tPA/plasminogen and ANGPTL4/8 combined almost completely reversed LPL inhibition by this set of proteins.

Very similar results were obtained for the LPL inhibitor APOC3, which is produced in the liver and belongs to a different class of proteins (21). In contrast, ANGPTL4/8-induced plasmin formation did not affect APOC2, a circulating LPL activator.

These data represent a major leap forward in our understanding of the role of ANGPTL8 in adipose tissue and indicate how the marked upregulation of ANGPTL8 during feeding enhances the local clearance of circulating triglycerides. By inactivating the above set of LPL inhibitors, adipocyte-derived ANGPTL8, *via* the formation of the ANGPTL4/8 complex, maintains LPL in adipose tissue in a fully active state after a meal, thereby allowing efficient storage of dietary triglycerides for future use. In contrast, in other tissues, which do not produce the ANGPTL4/8 complex, the liver-derived ANGPTL3/8 complex inhibits LPL.

As often with exciting new findings, the work also raises several new questions, mostly related to how the proposed new pathway plays out in vivo. An important next step would be to verify whether activated plasmin promotes cleavage of LPL inhibitors and thereby reduces plasma triglycerides in vivo. Furthermore, does ANGPTL4/8 promote plasmin-mediated cleavage of the LPL inhibitors in vivo? The first question could be addressed by modulation of the plasmin system in mouse models, for instance by genetically (in)activating endogenous plasmin inhibitors or administering synthetic small-molecule plasmin inhibitors. The second question could be addressed by examining whether the elevated plasma triglycerides in adipocyte-specific ANGPTL8-deficient mice can be abolished by activation or overexpression of plasmin (18).

An important additional question is where the interaction between ANGPTL4 and ANGPTL8 and especially between ANGPTL4/8 and tPA/plasminogen occurs. Although the ANGPTL4/8 complex has been measured in plasma (16), adipocyte-derived ANGPTL8 is not detectable in plasma (18), suggesting that the ANGPTL4/8 complex secreted by adipocytes may end up in the subendothelial space instead of the circulation. Based on that notion, it could be envisaged that the interaction between ANGPTL4/8 and tPA/plasminogen also happens in the subendothelial space, where there are binding sites for plasminogen and where plasmin is involved in the degradation of the extracellular matrix (22). But if plasmin is generated in the subendothelial space, how does it cleave liver-derived ANGPTL3/8 and APOC3 present in the capillaries?

Previous studies in human subjects have found correlations between plasma levels of components of the plasminogen system and plasma triglycerides (23, 24). The data presented in this article raise the possibility that these correlations are causal and that plasmin, and other factors that modulate plasmin activity, may influence plasma triglyceride levels in humans.

Overall, the observation that ANGPTL4/8 activates tPA and plasmin and promotes the cleavage of several LPL inhibitors gives important new insight into how after feeding, ANGPTL8 promotes LPL activity in adipose tissue and lowers triglyceride levels in plasma. The data underscore the distinct functions of ANGPTL8 as a partner with ANGPTL3 and ANGPTL4 and argue against the generic inactivation of ANGPTL8 as potential lipid-lowering therapy.

## Conflict of interest

The author declares no conflicts of interest with the contents of this article.
